# Anemia and chronic kidney disease are potential risk factors for mortality in stroke patients: a historic cohort study

**DOI:** 10.1186/1471-2369-11-27

**Published:** 2010-10-16

**Authors:** Patrizia Del Fabbro, Jean-Christophe Luthi, Emmanuel Carrera, Patrik Michel, Michel Burnier, Bernard Burnand

**Affiliations:** 1 Service of Neurology, Centre Hospitalier Universitaire Vaudois and University of Lausanne, Switzerland; 2 Institute of Social and Preventive Medicine (IUMSP), Centre Hospitalier Universitaire Vaudois and University of Lausanne, Switzerland; 3 Valais Health Observatory, Sion, Switzerland; 4 Service of Nephrology, Centre Hospitalier Universitaire Vaudois and University of Lausanne, Switzerland

## Abstract

**Background:**

Chronic kidney disease (CKD) is associated to a higher stroke risk. Anemia is a common consequence of CKD, and is also a possible risk factor for cerebrovascular diseases. The purpose of this study was to examine if anemia and CKD are independent risk factors for mortality after stroke.

**Methods:**

This historic cohort study was based on a stroke registry and included patients treated for a first clinical stroke in the stroke unit of one academic hospital over a three-year period. Mortality predictors comprised demographic characteristics, CKD, glomerular filtration rate (GFR), anemia and other stroke risk factors. GFR was estimated by means of the simplified Modification of Diet in Renal Disease formula. Renal function was assessed according to the Kidney Disease Outcomes Quality Initiative (K/DOQI)-CKD classification in five groups. A value of hemoglobin < 120 g/L in women and < 130 g/L in men on admission defined anemia. Kaplan-Meier survival curves and Cox models were used to describe and analyze one-year survival.

**Results:**

Among 890 adult stroke patients, the mean (Standard Deviation) calculated GFR was 64.3 (17.8) ml/min/1.73 m^2 ^and 17% had anemia. Eighty-two (10%) patients died during the first year after discharge. Among those, 50 (61%) had K/DOQI CKD stages 3 to 5 and 32 (39%) stages 1 or 2 (p < 0.001). Anemia was associated with an increased risk of death one year after discharge (p < 0.001). After adjustment for other factors, a higher hemoglobin level was independently associated with decreased mortality one year after discharge [hazard ratio (95% CI) 0.98 (0.97-1.00)].

**Conclusions:**

Both CKD and anemia are frequent among stroke patients and are potential risk factors for decreased one-year survival. The inclusion of patients with a first-ever clinical stroke only and the determination of anemia based on one single measure, on admission, constitute limitations to the external validity. We should investigate if an early detection and management of both CKD and anemia could improve survival in stroke patients.

## Introduction

Stroke is an increasing cause of mortality and severe neurological disability, including late-life dementia, worldwide [1-3]. Chronic kidney disease (CKD) is an independent risk factor for cerebrovascular diseases [4,5]. Anemia, a frequent feature of reduced kidney function, could actually play a role in the relationship between CKD and stroke [6]. However, the relationship between CKD-, anemia- and stroke-related outcomes needs to be studied further. Chronic kidney disease (CKD) is also an increasing public health problem worldwide, associated with poor outcomes and high cost [7]. A loss of renal function is associated with an elevated risk of stroke and cardiovascular diseases compared to the general population [8-10]. Other risk factors for stroke are commonly encountered in dialysis patients [11].

Friedman found out that serum creatinine is a strong and independent predictor of survival after a stroke [12], while another study indicated that this association was related to the presence of anemia [6]. When anemia was present with chronic kidney disease, the risk of stroke increased clearly compared to patients with CKD without anemia, in which the risk of stroke was only moderately increased. Among those patients with anemia and low creatinine clearance the crude stroke rate per 1000 person-years was 10.53 and, among those without anemia and low creatinine clearance, the rate was 2.85 [6]. To further study the role of CKD and anemia on outcome in stroke patients, the objective of this study was to examine the association between chronic kidney disease and anemia with in-hospital mortality and one-year survival among hospitalized stroke patients.

## Patients and Methods

### Setting, patients

In this retrospective cohort study, all consecutive patients hospitalized with a diagnosis of a first ever clinical stroke, and recorded in the Stroke Registry of the Neurology Service of an academic medical center in Western Switzerland, were included [13]. All patients discharged from the hospital after a hemorrhagic or ischemic stroke between 1^st ^January 2001 and 31^st ^December 2003 were considered for inclusion in a retrospective cohort study. We excluded patients who could not be reached at follow-up, especially those living abroad, refusals and cases with missing values for creatinine or hemoglobin, as well as subjects with incomplete coded discharge information.

### Data collection

Unless otherwise indicated, all information was retrieved from the Hospital Stroke Registry. This Stroke Registry was limited to patients with a first ever clinical stroke admitted to the Centre Hospitalier Universitaire Vaudois since 1979 [13]. With the exception of one-year survival, all information was collected during the index hospitalization for a first ever clinical stroke. Information on demographic characteristics and risk factors for stroke (i.e. hypertension, hypercholesterolemia, diabetes, and smoking habits (ex-, current or non-smoker)) were collected from the Stroke Registry. Other variables included the presence of ischemic heart disease and atrial fibrillation, peripheral arterial insufficiency (defined according to the clinical definition of claudication classified by Fontaine), a positive family history for stroke and/or heart disease and transient ischemic attack (TIA: a neurological disorder due to a temporary ischemia of the brain which lasts less than 24 hours). This information was prospectively asked to the patients or their family members at admission to the hospital and added to the stroke registry. Diabetes was considered present if fasting plasma glucose was > 7.0 mmol/l, a 2-hour value in the oral glucose tolerance test or a random plasma glucose concentration > 11.1 mmol/l, in the presence of symptoms [14]. Dyslipidemia was retained if LDL was > 2.6 mmol/l, or if, in absence of a LDL value, total cholesterol was > 5 mmol/l [15]. Patients with already treated diabetes and hypercholesterolemia were also registered. The presence of Ischemic heart disease was categorized as follows: none; Ischemic Heart disease (regrouping angina without heart attack and heart attack), and heart failure.

An evaluation of the functional status was also collected from the Registry. The functional status is a disability scale that represents the activities of daily living in a five-level scale with Stage I representing no disability, Stage II mild disability (return to all activities, although with difficulty), Stage III moderate disability (return to main activities, although with difficulty), Stage IV severe disability (impossible to return to most activities) and Stage V death. We did not use the widely used modified Rankin Scale (mRS) because our five-level scale was used since the creation of the Lausanne Stroke Registry (LSR) in 1978. At this time, the mRS had not yet been defined. In comparison, Stage I (LSR) corresponds to Stage 0-I (mRS), Stage II to Stage II, Stage III to Stage III-IV, Stage IV to Stage V and Stage V to Stage VI. The serum creatinine, hemoglobin and hematocrit values were collected from the electronic files of the hospital's laboratory. The first hemoglobin and serum creatinine values of the hospitalization were used for each patient. Anemia was defined as a hemoglobin level < 120 g/L in women and < 130 g/L men according to the World Health Organization (WHO) definition [16]. We estimated the Glomerular Filtration Rate (GFR) by means of the simplified Modification of Diet in Renal Disease formula [17,18]. Renal function was assessed according to the Kidney Disease Outcomes Quality Initiative (K/DOQI)-CKD classification in five groups [17]. Stage I corresponded to patients with GFR values ≥ 90 ml/min/1.73 m^2^, Stage II to GFR values from 60 to 89 ml/min/1.73 m^2^, Stage III to GFR values from 30 to 59 ml/min/1.73 m^2^, Stage IV to GFR values from 15 to 29 ml/min/1.73 m^2^, and Stage V to GFR values < 15 ml/min/1.73 m^2^. The Stroke Registry number allowed us to access to the medical chart for each stroke patient hospitalized between 2001 and 2003, featuring all necessary information about the patients, such as interventions, reports of admission notes and medical consultations and discharge letters, discharge status including death, and discharge diagnoses (International Classification of Diseases 10^th ^Revision (ICD-10)). The Charlson comorbidity index, a weighted average of selected comorbidities, was calculated by means of administrative data, using an algorithm that was recently developed for ICD-10 codes [19].

### Main outcome measures

The outcomes of interest were in-hospital mortality and one-year survival in hospitalized stroke patients. Follow-up information to establish survival after discharge from hospital was collected via telephone interviews with the patients, their relatives, their physicians, and/or the nursing homes. For a few patients, this information was obtained from Population Registries. The one-year mortality rate was calculated from the date of discharge from hospital.

### Statistical analyses

Bivariate analyses, including chi-square tests, Fisher's exact tests, Student T-tests or ANOVA methods, where appropriate, were used in a first step. Kaplan-Meier (KM) survival curves were obtained using the log rank test to assess if KM survival curves for patients with or without CKD or with different levels of hemoglobin were statistically different. For these survival curves, the follow-up was extended up to over five years, to a maximum of 1889 days. A univariate survival analysis was performed, using hemoglobin and GFR as continuous variables (Cox proportional hazard analysis).

We then conducted multivariate analyses adjusted for potential confounding factors. Cox models were used to assess one-year survival and to calculate adjusted hazard ratios with their associated 95% confidence intervals [20]. We checked the proportional hazard assumption. Our modeling strategy was based on a priori consideration of bivariate analyses' results and clinical judgment to select the more important variables. We applied the Mickey and Greenland method to select the final model, using a step-forward regression method with a p-value for entry of 0.20 [21]. Variables included in the first model were hemoglobin, GFR, age, gender, hypertension, diabetes, past and current smokers (reference = non smokers), hypercholesterolemia, heart failure, ischemic heart disease, peripheral arterial insufficiency, atrial fibrillation, TIA before the stroke attack, functional status in four categories (reference = functional status I), and the Charlson comorbidity index in four categories (reference = Charlson score 0 or 1). A significance level of 0.05 was used in all statistical analyses done in this study. All analyses were performed with the SAS Software, version 9.1 (SAS Institute, Cary, NC, USA).

The collection and use of the data was authorised by the Federal Data Protection and Information Commissioner within the framework of the Data Protection Law. Every included patient gave informed consent.

## Results

### Baseline Characteristics

A total of 963 eligible patients aged 16 to 97 were considered. We excluded two patients who had a transient ischemic attack and not a stroke, and three patients who had already had a previous symptomatic stroke. In addition, 10 subjects who could not be reached by phone, five who denied participation and 20 patients living abroad were excluded, as well as 16 with missing laboratory values for creatinine or hemoglobin, 10 discharged in 2004, and seven because of missing ICD-10 diagnostic codes. Among the 890 patients included, the mean (standard deviation: SD) age was 69.0 (15.7) years (25^th ^to 75^th ^interquartile range 59-80); 54% were men. A history of hypertension was present in 59% of patients, 15% had diabetes, 39% hypercholesterolemia, and 22% were current smokers. A history of cardiovascular disease was reported by 41% of the patients, a family history of stroke or coronary artery disease by 2%, migraine by 2%, TIA before stroke by 9%, and peripheral arterial insufficiency by 4% (Table 1, columns 1 and 2). The mean (SD) Charlson comorbidity index was 2.8 (1.6).

**Table 1:** Hospital and One-Year Mortality Related to Patients Characteristics and Risk Factors for Stroke, CHUV, 2001-2003, N = 890 on admission and N = 857 at one year

Characteristic and risk factors for stroke	Number (%) N (%)	Hospital mortality N (%)	P value	One-Year mortality N (%)	P value
**N (%)**		33 (3.7)		82 (9.6)	
**Age**			< 0.001		< 0.001
< 60 years	240 (27.0)	2 (6.1)		6 (7.3)	
60-70 years	151 (17.0)	1 (3.0)		6 (7.3)	
71-80 years	267 (30.0)	11 (33.3)		26 (31.7)	
< 80 years	232 (26.0)	19 (57.6)		44 (53.7)	
**Gender**			0.21		0.20
Male	479 (53.8)	14 (42.4)		39 (47.6)	
Female	411 (46.2)	19 (57.6)		43 (52.4)	
***Risk Factors for Stroke***					
**Hypertension**	528 (59.3)	19 (57.6)	0.86	49 (59.8)	1.00
**Diabetes**	133 (15.0)	3 (9.1)	0.46	17 (20.7)	0.15
**Hypercholesterolemia**	346 (38.9)	6 (18.2)	0.02	19 (23.2)	0.001
**Smoking**			0.009		0.002
No	595 (66.8)	30 (90.9)		67 (81.7)	
Yes	198 (22.2)	3 (9.1)		6 (7.3)	
Ex-smoker	97 (11.0)	0		9 (11.0)	
***Antecedent***					
**Ischemic Heart Disease**	126 (14.2)	7 (21.2)	0.30	15 (18.3)	0.24
**Heart failure**	97 (11.0)	8 (24.2)	0.02	20 (24.4)	< 0.001
**Peripheral Arterial Insufficiency**	36 (4.0)	0	0.64	9 (11.0)	0.005
**Atrial Fibrillation**	125 (14.0)	9 (27.3)	0.04	16 (19.5)	0.12
**Positive Family History for Stroke/Heart Disease**	16 (1.8)	0	1.00	0	0.34
**TIA Before Stroke**	77 (8.7)	2 (6.1)	1.00	2 (2.4)	0.04
***Severity of disease***					
**Functional Status**			< 0.001		**<**0.001
I	263 (29.6)	0		6 (7.3)	
II	201 (22.6)	0		10 (8.2)	
III	164 (18.4)	0		13 (15.9)	
IV	230 (25.8)	1 (3.0)		53 (64.6)	
Death	32 (3.6)	32 (97.0)			
**Charlson Comorbidty Index**			0.62		< 0.001
Charlson score 0 or 1	268 (30.1)	7 (21.2)		11 (13.4)	
Charlson score 2	93 (10.5)	3 (9.1)		9 (11.0)	
Charlson score 3	298 (33.5)	12 (36.4)		24 (29.3)	
Charlson score ≥4	231 (26.0)	11 (33.3)		38 (46.3)	
***Chronic Kidney Disease***					
Stage 1 (GFR ≥90)	59 (6.3)	0	0.052	5 (6.1)	< 0.001
Stage 2 (GFR: 60-89)	469 (52.7)	13 (39.4)		27 (32.9)	
Stage 3 (GFR: 30-59)	335 (37.6)	18 (54.6)		43 (52.4)	
Stage 4 or 5 (GFR ≤29)	27 (3.0)	2 (6.1)		7 (8.5)	
**Mean (SD) GFR ml/min/1.73 m**^**2**^	64.3 (17.8)	55.2 (12.6)	< 0.001	55.6 (20.6)	< 0.001

TIA: Transient Ischemic Attack

GFR: Glomerular Filtration Rate ml/min/1.73 m^2^

### Prevalence of CKD

The mean (SD) serum creatinine value was 102.7 (43.3) μmol/L (25^th ^to 75^th ^interquartile range: 83 to 109 μmol/L). The mean (SD) calculated GFR was 64.3 (17.8) ml/min/1.73 m^2 ^(25^th ^to 75^th ^interquartile range: 53.7 to 76.2 ml/min/1.73 m^2^). The distribution of renal impairment according to K/DOQI CKD stages among the entire cohort showed that 59 (7%), 469 (53%), 335 (38%), 21 (2%) and 6 (1%) of the patients had Stages I to V GFR respectively. The mean GFR was statistically lower in elderly patients, in women, in patients with hypertension, diabetes, a history of heart diseases, heart failure, and a family history for stroke (Table 2).

**Table 2:** Chronic Kidney Disease Prevalence and Mean Glomerular Filtration Rate according to Patients Characteristics and Risk Factors for Stroke, CHUV, 2001-2003, N = 890

Characteristic and risk factors for stroke	Number N (%)	Chronic Kidney Disease N (%)				P value	Mean (SD) GFR ml/min/1.73 m2	P value
		I	II	III	IV-V			
**N (%)**	890 (100.0)	59 (6.6)	469 (52.7)	335 (37.6)	27 (3.0)		64.3 (17.8)	
**Age**						< 0.001		< 0.001
≤60 years	240 (27.0)	39 (16.3)	177 (73.8)	20 (8.3)	4 (1.7)		76.8 (16.5)	
60-70 years	151 (17.0)	10 (6.6)	92 (60.9)	47 (31.1)	2 (1.3)		67.4 (16.2)	
71-80 years	267 (30.0)	8 (3.0)	121 (45.3)	127 (47.6)	11 (4.1)		59.1 (15.8)	
< 80 years	232 (26.0)	2 (0.86)	79 (34)	141 (61)	10 (4.3)		55.2 (13.9)	
**Gender**						< 0.001		< 0.001
Male	479 (53.8)	52 (10.9)	287 (59.9)	126 (26.3)	14 (2.9)		69.1 (18.7)	
Female	411 (46.2)	7 (1.7)	182 (44.3)	209 (50.9)	13 (3.2)		58.6 (14.8)	
***Risk Factors for stroke***								
**Hypertension**	528 (59.3)	22 (4.2)	246 (46.6)	239 (45.3)	21 (3.9)	< 0.001	60.3 (16.9)	< 0.001
**Diabetes**	133 (15.0)	11 (8.3)	51 (38.3)	64 (48.1)	7 (5.3)	0.003	59.6 (19.7)	0.003
**Hyper-Cholesterolemia**	346 (38.9)	20 (5.8)	181 (52.3)	135 (39)	10 (2.9)	0.812	63.7 (17.0)	0.47
**Smoking**						< 0.001		< 0.001
No	595 (66.8)	24 (4.03)	285 (48)	268 (45.04)	18 (3.03)		61.1 (16.2)	
Yes	198 (22.2)	30 (15.15)	127 (64.1)	37 (18.7)	4 (2.02)		73.7 (18.0)	
Ex-smoker	97 (11.0)	5 (5.15)	57 (58.8)	30 (31)	5 (5.15)		64.7 (19.8)	
***Antecedent***								
**Ischemic Heart Disease**	126 (14.2)	3 (2.4)	55 (43.7)	62 (49.2)	6 (4.8)	0.005	59.2 (16.1)	< 0.001
**Heart failure**	97 (11.0)	3 (3.1)	35 (36.1)	54 (55.7)	5 (5.2)	0.0003	55.4 (17.6)	< 0.001
**Peripheral Arterial Insufficiency**	36 (4.0)	2 (5.6)	18 (50)	14 (38.9)	2 (5.6)	0.703	62.4 (21.4)	0.52
**Atrial Fibrillation**	125 (14.0)	4 (3.2)	51 (40.8)	64 (51.2)	6 (4.8)	0.0017	57.0 (16.8)	< 0.001
**Positive Family History for Stroke/Heart Disease**	16 (1.8)	4 (25)	11 (68.8)	1 (6.25)	0	0.005	79.5 (15.0)	< 0.001
**TIA before Stroke**	77 (8.7)	5 (6.5)	39 (50.7)	31 (40.3)	2 (2.6)	0.964	62.0 (17.3)	0.25
***Severity of disease***								
**Functional Status**						0.006		0.04
I	263 (29.6)	11(4.2)	157 (59.7)	92 (35)	3 (1.14)		65.3 (15.6)	
II	201 (22.6)	20 (9.9)	104 (51.7)	71 (35.3)	6 (3)		65.2 (17.8)	
III	164 (18.4)	10 (6.1)	91 (55.5)	54 (33)	9 (5.5)		65.1 (19.3)	
IV	230 (25.8)	18 (7.8)	104 (45.2)	100 (43.5)	8 (3.5)		62.7 (19.4)	
Death	32 (3.6)	0	13 (40.6)	18 (56.3)	1 (3.13)		56.2 (11.6)	
**Charlson score**						< 0.001		< 0.001
Charlson score 0 or 1	268 (30.1)	18 (6.7)	174 (65)	74 (27.6)	2 (0.8)		68.8 (16.2)	
Charlson score 2	93 (10.5)	2 (2.2)	47 (50.5)	42 (45.2)	2 (2.2)		62.2 (14.4)	
Charlson score 3	298 (33.5)	23 (7.7)	156 (52.4)	111 (37.3)	8 (2.7)		64.7 (17.4)	
Charlson score ≥4	231 (26.0)	16 (6.9)	92 (39.8)	108 (46.8)	15 (6.5)		59.2 (19.9)	

TIA: Transient Ischemic Attack

GFR: Glomerular Filtration Rate ml/min/1.73 m^2^

### Prevalence of Anemia

**Figure 1:**
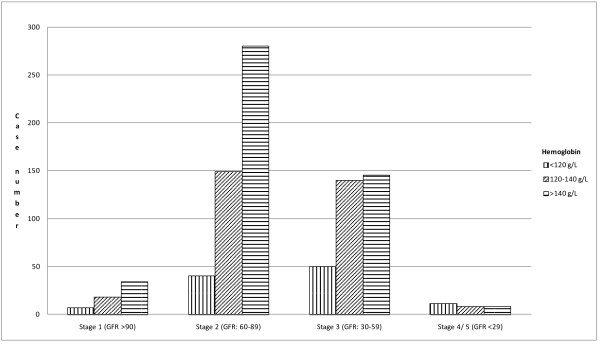
**Frequencies of Patients Hospitalised for a First Stroke, by Hemoglobin Level (< 120, 120-140, > 140 g/l), according to K/DOQI CKD Stage**.

The mean (SD) hemoglobin at admission was 139 (18) g/L (25^th ^to 75^th ^interquartile range 129-152 g/L). Hemoglobin < 140 g/L was found in 53% of the patients, 35% had hemoglobin between 120 g/L and 140 g/L and 12% < 120 g/L. Anemia was present in 148 patients (17%). Figure 1 depicts the frequencies of stroke patients by categories of hemoglobin, according to K/DOQI CKD stages. Among patients with hemoglobin < 120 g/L, a lower proportion of K/DOQI CKD Stages I or II was observed compared to patients with hemoglobin > 140 g/L (Table 3). The mean GFR was progressively lower with an increasing severity of anemia (Table 3) across the three hemoglobin categories. Anemia was present in 67 patients (45%) with K/DOQI CKD Stages I or II and 81 (55%) with K/DOQI CKD Stages III to V (p < 0.0001).

**Table 3:** Hemoglobin Levels Related to CKD, GFR and Mortality in Patients Hospitalized for a First Stroke, CHUV, 2001-2003, N = 890

	Hemoglobin in g/L				
N (%)	< 120 g/L N (%) or Mean (SD)	120-140 g/L N (%) or Mean (SD)	>**140 g/L N (%)** or Mean (SD)	Total (%)	P Value
**N = 890**	108 (12.1)	315 (35.4)	467 (52.5)	890 (100)	
**Gender**					< 0.001
**Male**	42 (38.9)	123 (39.1)	314 (67.2)	479 (53.8)	
**Female**	66 (61.1)	192 (60.1)	153 (32.8)	411 (46.2)	
**Age**					< 0.001
≤**60 years**	20 (18.5)	60 (19.1)	160 (34.3)	240 (27.0)	
**60-70 years**	14 (13.0)	44 (14.0)	93 (19.9)	151 (17.0)	
**71-80 years**	31 (28.7)	102 (32.4)	134 (28.7)	267 (30.0)	
**< 80 years**	43 (39.8)	109 (34.6)	80 (17.1)	232 (26.0)	
**Mean (SD) Hemoglobin**	10.7 (12.7)	13.1 (5.4)	15.2 (10.1)	13.9 (1.8)	< 0.001
**CKD**					
Stage 1 (GFR ≥90)	7 (6.5)	18 (5.7)	34 (7.3)	59 (6.6)	< 0.001
Stage 2 (GFR: 60-89)	40 (37.0)50 (46.3)	149 (47.3)	280 (60.0)	469 (52.7)	
Stage 3 (GFR: 30-59)	11 (10.2)	140 (44.4)	145 (31.1)	335 (37.6)	
Stage 4/5 (GFR ≤29)		8 (2.5)	8 (1.7)	27 (3.0)	
**Mean (SD) GFR ml/min/1.73 m**^**2**^	56.7 (21.6)	62.6 (16.8)	67.2 (16.8)	64.3 (17.8)	< 0.001
**In-hospital mortality (N = 890)**	6 (5.6)	14 (4.4)	13 (2.8)	33 (3.7)	0.27
**One-year mortality (N = 857)**	23 (22.6)	30 (10.0)	29 (6.4)	82 (9.6)	< 0.001

GFR: Glomerular Filtration Rate ml/min/1.73 m^2^

### Survival

**Figure 2:**
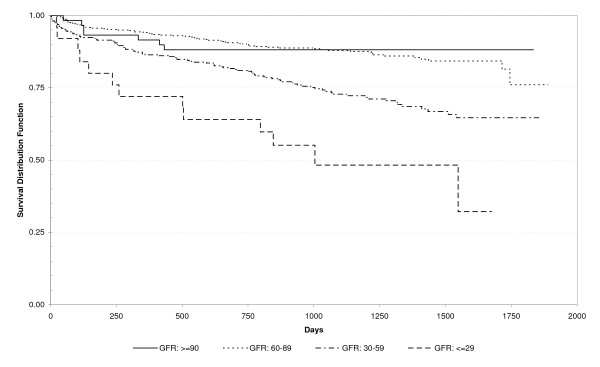
**Kaplan-Meier Survival Curve According to K/DOQI CKD Stage, among Patients Hospitalized for a First Stroke, N = 856 [GFR (ml/min/1.73 m**^**2**^**) ≥90: 59; GFR 60-89: 456; GFR: 30-59: 316; GFR ≤29: 25)]**.

**Figure 3:**
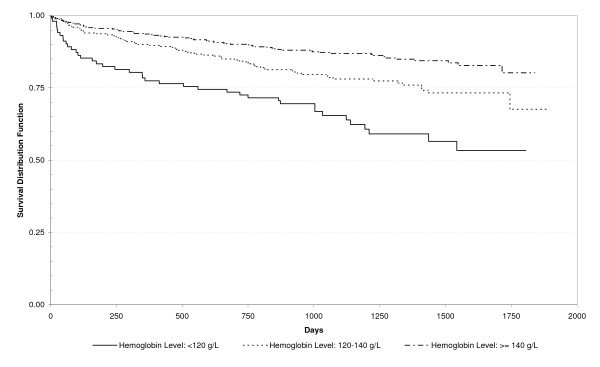
**Kaplan-Meier Survival Curve for Individuals with Hemoglobin Levels of < 120 (102), 120-140 (300) and > 140 (454) g/L, among Patients Hospitalized for a First Stroke, N = 856**.

Thirty-three (3.7%) patients died during their hospitalization. Among them, 20 (61%) had K/DOQI CKD Stages III to V and 13 (39%) Stages I or II (p = 0.052), and their mean GFR was 55.2 ml/min/1.73 m^2^. Eighty-two (10%) patients died during the first year of follow-up. Among them 50 (61%) had K/DOQI CKD Stages III to V and 32 (39%) Stages I or II (p < 0.0001), and their mean GFR was 55.6 ml/min/1.73 m^2^. Indeed, the presence of an increasing stage of CKD was associated with an increased risk of death during the hospitalization and the first year after discharge (Table 1). Anemia was also associated with an increased risk of death (Table 3). Proportions of in-hospital and one-year mortality increased progressively, with an increasing degree of anemia expressed in the three hemoglobin categories (Table 3). Kaplan-Meier survival analyses indicated that K/DOQI CKD Stages III and IV (Figure 2) and lower hemoglobin (Figure 3) were risk factors for decreased survival, log rank test p < 0.001 and p < 0.001, respectively. In univariate survival analysis, both hemoglobin and GFR were positively associated with survival, as indicated by hazard ratios of 0.976 (95% CI 0.966-0.986) and 0.970 (95% CI 0.959-0.982), respectively.

### Multivariable Analyses

Cox models were used to assess the determinants of survival at one year. Hemoglobin was independently associated with survival at one-year (Table 4) controlling for age, glomerular filtration rate, hypertension, hypercholesterolemia, previous TIA, functional status and Charlson comorbidity index. For each 10 g/L increase in hemoglobin, the annual subsequent probability of death declined by 1.6%. GFR was not statistically associated with survival in the model. The latter relation was not statistically significant, however. The interaction term between hemoglobin and GFR was also not significantly associated with survival.

**Table 4:** Results of Cox Proportional Hazard Model for One-Year Mortality in Patients Hospitalized for a First Stroke, CHUV, 2001-2003, N = 856

Variables	Parameter Estimate	Standard Error	Hazard Ratio (95% CI)
**Hemoglobin (g/L, continuous)**	- 0.0163	0.0056	0.98* (0.97-1.00)
**Glomerular filtration rate (ml/min/1.73 m**^**2**^**, continuous)**	- 0.0114	0.0073	0.99 (0.98-1.01)
**Age (years, continuous)**	0.0398	0.0113	1.04* (1.02-1.07)
**Hypertension**	- 0.3911	0.2359	0.68 (0.43-1.07)
**Hypercholesterolemia**	- 0.4503	0.2728	0.64 (0.37-1.09)
**Previous transitory ischemic attack**	- 1.1605	0.7230	0.31 (0.08-1.29)
**Functional status II (ref = I)**	0.5474	0.5196	1.73 (0.62-4.79)
**Functional status III (ref = I)**	0.9331	0.5008	2.54 (0.95-6.79)
**Functional status IV (ref = I)**	1.8335	0.4471	6.26* (2.61-15.02)
**Charlson comorbidity index 2 (ref = 0 or I)**	0.4103	0.4564	1.51 (0.62-3.69)
**Charlson comorbidity index 3 (ref = 0 or I)**	0.0646	0.3743	1.07 (0.51-2.22)
**Charlson comorbidity index 4 (ref = 0 or I)**	0.6228	0.3662	1.86 (0.91-3.82)

* p < 0.05

## Discussion

We observed that anemia and chronic kidney disease are two frequent conditions among stroke patients. Anemia was independently associated with a decreased one-year survival. Although GFR was associated with mortality in bivariate analyses, this relationship was not statistically significant in multivariate analyses. These associations between reduced kidney function and anemia and the risk of death among stroke patients have not been reported previously.

So far, most studies have concentrated on the risk of death among heart failure and myocardial infarction patients with CKD and anemia [22-24]. Previous studies already showed that CKD [25,26] and anemia [27,28] increase the risk of death among patients with coronary heart disease, heart failure and ischemic heart disease. In this study we wanted to assess if we observed similar findings for stroke patients. Previous studies have shown that an elevated serum creatinine concentration is associated with an increased risk of stroke and that CKD and anemia are risk factors for stroke [6,29]. Indeed, 41% of our stroke study patients were in K/DOQI CKD stages III to V and 17% had anemia.

Our study findings were similar to those obtained by Friedman, in which serum creatinine was found to be a strong and independent predictor of survival after stroke in the elderly [12]. In our study, elevated serum creatinine was associated with increased hospital mortality and decreased one-year survival.

Anemia is common among individuals with reduced kidney function, generally because of a decreased production of erythropoietin [25-31]. It is a consequence of chronic kidney disease and it also worsens the progression of renal insufficiency. Anemia increases the prevalence of CKD with decreasing creatinine clearance [32,33]. In our study, the mean GFR was lower in patients with anemia and more patients in K/DOQI CKD Stages III to V had anemia. Anemia in patients with CKD may predispose to ischemic heart disease, heart failure and premature death [22,34]. Previous studies reported an increased risk of death among anemic patients with or without renal impairment [35]. In our study, among stroke patients with anemia, the hospital and one-year mortality rates increased with a decreasing hemoglobin.

There are different reasons why anemia and chronic kidney disease could be risk factors for mortality: chronic anemia involves hemodynamic compensations, for example an increase in heart rate, cardiac index and stroke work, as well as an amplification of hypoxia. These compensations stress the ventricular function and worsen the dysfunction among patients with heart failure and, thus, could consequently increase the risk of death [36]. Anemia is associated with changes in left ventricular anatomy among patients with CKD [37], a change that could also increase death rates. Furthermore, reduced hemoglobin may be associated with other risk factors of cardio-vascular mortality [29] and it may result in worsening ischemia by amplifying hypoxia [37]. Several studies have demonstrated that the level of kidney function is a significant risk factor for all cause mortality in patients with heart failure and hypothesized several explanations [29]. For example, reduced kidney function may be associated with other risk factors for mortality. In addition, kidney disease may be a risk factor for the progression of cardiac dysfunction by worsening fluid and sodium retention, thus fostering left ventricular dilatation and hypertrophy, a strong predictor of stroke risk [38]. Furthermore CKD may induce other pathophysiologic processes that may increase stroke risk, like inducing oxidative stress, which could promote atherosclerosis. These elements may contribute to cerebral ischemia and increase the risk of ischemic stroke [39]. Moreover, risk factors for stroke, such as diabetes, hypertension or cardiovascular diseases are more common among dialysis patients [6] and uremia causes accelerated atherosclerotic vascular disease. One study showed that increased aortic stiffness among CKD patients was an explanation why CKD is a predictor of cardio- and cerebrovascular mortality [40,41].

Most patients with ESRD and even less severe kidney dysfunction are anemic because of a deficient erythropoietin production. Erythropoietin was shown to have neuronal protective effects in animal models of ischemia-induced stroke and brain injury [42]. When renal function is reduced, erythropoietin production is impaired, which could limit erythropoietin-induced neuronal protection against anemia-induced stroke. Thus, the combination of anemia and CKD could interact to make a stroke event more likely to occur or more likely to be clinically severe [6]. Furthermore, it was shown that hemodialysis patients have better outcomes than patients suffering from renal insufficiency but getting no treatment. However, patients on continuous ambulatory peritoneal dialysis have a greater risk of stroke than the general population. This is explainable by a poor control of hypertension, which is presumably partly due to over-hydration in patients getting peritoneal dialysis [43].

This study has several limitations. First, it included only patients with first-ever clinical stroke, and therefore did not reflect the impact of CKD and anemia on all patients suffering from stroke. However, compared with all patients suffering from stroke, our cohort represents a more homogenous population, with younger patients in which secondary prevention and treatment may have higher impact. Second, we had no information on the duration of CKD or anemia. We did not know the cause of anemia for all patients. For some patients, anemia could be linked to CKD. There could be other causes of anemia like folate, iron, vitamin B12 deficiency, gastro-intestinal diseases, -bleeding, cancer or chronic inflammatory illness. Third, we did not know the cause of death of the stroke patients. We did not know whether they died from complications of their stroke or from another cause. Fourth, we took all the laboratory values from the first day of admission to hospital. Maybe these values were wrongly elevated in patients who arrived in the hospital in a dehydrated situation. Finally, we may question the study's external validity because it was conducted in an academic medical centre. However, this centre is also the principal referral centre for the region from which most patients were referred.

## Conclusion

This study has confirmed the deleterious effect of renal deficiency and anemia on survival in patients who sustained a first stroke. We have shown that anemia is independently associated with a lower one-year survival. Thus, it might be useful to treat patients with kidney disease and anemia as early as possible in order to reduce the complications and co-morbidities that result from these diseases. Indeed, CKD is very often under diagnosed and under-treated [12]. Appropriate trials should be conducted to examine if early interventions to detect and treat systematically renal insufficiency and anemia in patients experiencing a first stroke may improve survival and quality of life. However, the solution may not be easy. Indeed, a trial of darbepoetin alfa in patients with type 2 diabetes and chronic kidney disease, not undergoing dialysis, (TREAT study) has indicated an increased risk of stroke [43].

## Declaration of Competing interests

The authors declare that they have no competing interests.

## Authors' contributions

PDF performed the statistical analyses, interpreted the data and was involved in the drafting of the manuscript.

J-ChL conceived and designed the study, analysed and interpreted the data, was involved in the drafting of the manuscript and revised it critically for important intellectual content.

EC participated to the acquisition and interpretation of data, and revised the manuscript critically.

PM participated to the conception and design of the study, to data acquisition, interpreted the results and revised the manuscript critically.

MB participated to the conception and design of the study, interpreted the results and revised the manuscript critically.

BB participated to the conception and design of the study, interpreted the results and revised the manuscript critically. He is the guarantor of the work.

All authors read and approved the final manuscript.

## Pre-publication history

The pre-publication history for this paper can be accessed here:

http://www.biomedcentral.com/1471-2369/11/27/prepub
